# Evaluation of efficiency and safety of combined montelukast sodium and budesonide in children with cough variant asthma: A protocol for systematic review and meta-analysis

**DOI:** 10.1097/MD.0000000000026416

**Published:** 2021-06-25

**Authors:** Qiongyao Tang, Huizhen Lei, Jinbing You, Jiangjiang Wang, Junyi Cao

**Affiliations:** a Department of Pediatrics, the First People's Hospital of Jiangxia District; b Department of Pediatrics, Hubei Maternal and Child Health Care Hospital, Wuhan 430200, Hubei, PR China.

**Keywords:** budesonide, child, cough variant asthma, efficacy, montelukast sodium

## Abstract

**Background:**

Cough variant asthma (CVA) is classified as a distinct form of asthma. As the primary or only symptom, cough is the leading cause for the most prevalent chronic cough among kids. The American College of Clinical Pharmacy, British Thoracic Society, and Chinese guidelines established for diagnosing and treating chronic cough in kids recommend inhaled corticosteroids, combined with leukotriene receptor antagonists when necessary.

**Methods:**

We will conduct a comprehensive search in major databases using keywords to find studies related to the analysis of montelukast sodium and budesonide for treating CVA in kids. Two reviewers will independently assess the quality of the selected research articles and perform data extraction. Next, we will use the RevMan software (version: 5.3) to conduct the statistical analysis of the present study.

**Results:**

This study will assess the efficacy and safeness of using montelukast sodium and budesonide to treat kids with CVA by pooling the results of individual studies.

**Conclusion:**

Our findings will provide vigorous evidence to judge whether montelukast sodium and budesonide therapy is an efficient form of therapy for CVA patients.

**Ethics and dissemination:**

Ethics approval is not needed for the present meta-analysis.

**OSF registration number:**

May 17, 2021.osf.io/cuvjz (https://osf.io/cuvjz/).

## 1 Introduction

Cough variant asthma (CVA) is classified as a distinct form of asthma. It is primarily triggered by mast cells, eosinophils, T lymphocytes, and similar types of inflammatory cells associated with inflammation in the airway.^[1,2]^ CVA is also a widespread subgroup of bronchial asthma, which is extremely prevalent among kids.^[3,4]^ In CVA patients, cough is the only or primary clinical manifestation, particularly during the night,^[5,6]^ but bronchodilator treatment is effective. Currently, the first-line of treatment for CVA include methods identical to the ones used to treat asthma, primarily consisting of antihistamines, inhaled bronchodilator, glucocorticoid drugs, and leukotriene receptor antagonist.^[7,8]^

However, long-term high-dose hormone inhalation can lead to systemic adverse hormone reactions and higher likelihood of relapsing after 1 stops using the drug.^[9]^ The best form of treatment consists of a lasting therapeutic effect and usage of systemic and local administration of immune regulators over a period exhibits side effects associated with corticosteroids. Generally, patients and their families show reluctancy to use glucocorticoid hormones therapy.^[10,11]^ Montelukast sodium refers to a potent selective cysteinyl leukotriene 1 receptor (CysLT1) antagonist, which competitively blocks the pro-inflammatory effect of CysLTs. CysLTs are known to have a positive correlation with allergic rhinitis and pathophysiology of asthma.^[12]^ Besides, CysLTs lead to bronchodilation through the creation of beta-2-stimulating medication.^[13]^ Moreover, glucocorticoid cannot inhibit the synthesis and release of CysLTs and is ineffective against the anti-inflammatory effect. Montelukast is a compound that contains anti-inflammatory characteristics, and it is orally active. Montelukast can effectively improve medical indicators of asthmatic inflammation (such as respiratory functionality and forced expiratory volume within 1 second [FEV1] in clinical trials).

## 2 Objectives

The present meta-analysis refers to the weighted combination research method which involves the integrated evaluation of multiple published research results under a single research topic. Its application can greatly improve the efficacy of statistical tests, solve inconsistencies in research results, and generalise research conclusions to help the general population. In this study, meta-analysis was used to strictly analyze and/or evaluate the design quality of clinical trials related to the use of montelukast sodium and budesonide to treat CVN, and to methodically assess the clinical efficacy and safeness of using montelukast sodium and budesonide to treat CVN. The authors hope to provide better guidance for medical practices.

## 3 Materials and methods

The current methodical review and meta-analysis is registered with OSF (https://osf.io/) and will adhere to the established guidelines for Preferred Reporting Items for Systematic Reviews and Meta-Analyses (PRISMA).^[14]^

### 3.1 Inclusion criteria for literature

(1) Selected studies must be Controlled Randomized Trials assessing the efficacy and safety of using montelukast sodium and budesonide to treat CVA.
(2) The patients who satisfy the clear diagnostic criteria of CVA (international or domestic) are not more than 14 years old, regardless of gender.
(3) Intervention measures: the control set was administered budesonide aerosol inhalation alone or montelukast sodium alone or placebo or other intervention method. The experimental set was administered montelukast sodium in combination with budesonide aerosol inhalation; the dosage is not limited; the course of treatment was more than 8 weeks.
(4) There are consistent evaluation indicators and judgment indicators of results: which includes peak expiratory flow, forced vital capacity, FEV1, recurrence rate, incidence of adverse reactions, total effective rate, biochemical indicators, marked effective rate, etc.
(5) The publication language of selected studies was restricted to English and Chinese.

### 3.2 Literature exclusion criteria

(1) Studies with the same test results published several times;
(2) Studies containing obvious errors in statistical analysis;
(3) Failure to provide sufficient original data and obtain useless data;
(4) Research on animal experiments.

### 3.3 Literature retrieval

**Figure 1:**
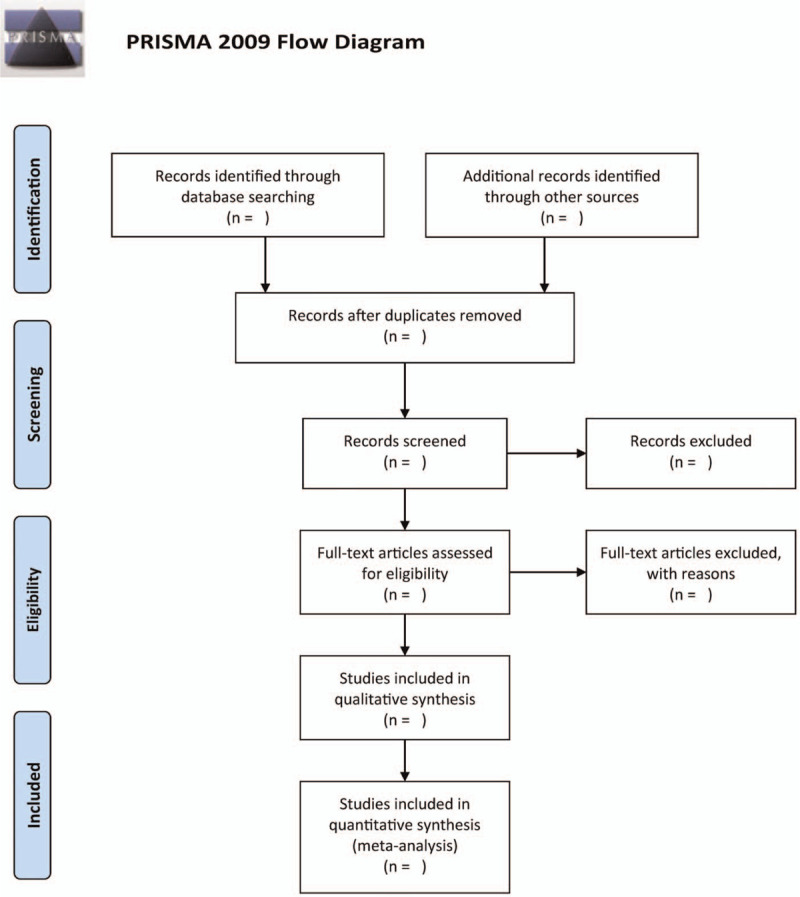
The research flowchart.

A comprehensive search will be performed on the following electronic databases: Web of Science, PubMed, The Cochrane Library, WanFang database, Chinese National Knowledge Chinese biomedical literature database (CBM), Chinese National Knowledge Infrastructure will be searched using a computer from inception till May 17, 2021. The following keywords will be used for the search: montelukast, budesonide, cough variant asthma, child. Figure 1 illustrates the study selection process.

### 3.4 Outcome assessment indicators

(1) Total clinical response rate;
(2) recurrence rate;
(3) lung function: FEV1, FEV1%, FEV, and peak expiratory flow.

### 3.5 Risk bias and quality assessment

A couple of independent reviewers will perform study screening, evaluate the systematic quality, perform data extraction, and crosscheck the studies. During all disagreements, a third researcher will be asked to help decide whether to include the studies. The methodological quality of each included study will be based on the bias risk assessment of Cochrane Collaboration. The data extraction includes the name of the first author, published date, sample size, age, intercession measures, course of treatment, follow-up time, and outcome indicators.

### 3.6 Statistical analysis

All statistical and meta-analysis will be conducted using the RevMan software (version: 5.3). The relative risk and 95% confidence interval will be used as the effect index for the binary variable data, and the standard mean difference and 95% confidence interval is used as the effect index for the measurement data, *P* ≥ .05 indicated statistical significance. If there is heterogeneity in the results, the random effect model is adopted for analysis; else, a fixed effect model is used.

## 4 Discussion

CVA is classified as a distinct form of asthma, and it has a similar pathogenesis to that of typical asthma. Both conditions are caused by immune, genetic and environmental factors, such as chronic airway inflammation, airway hyperresponsiveness, allergen sensitization, and airway remodelling. Recently, cysteinyl leukotrienes (CysLTs) have been used as critical inflammatory mediators in vivo.^[15]^ An increasing number of studies show that CysLTs have an active part in the incidence and development of CVA. According to major guidelines at home and overseas, inhaled low-dose glucocorticoids combined with bronchodilators is widely recommended as treatment for CVA.^[16]^ However, inhaled high-dose glucocorticoids can be absorbed from the lung, which directly leads to systemic adverse reactions. Gina indicated that the effect of adding a control drug is better than simply increasing the dose of hormone.^[16]^ The use of Cyslt1rs antagonists combined with inhaled corticosteroids is recommended for patients with poor control.^[15]^ Therefore, the present meta-analysis aims to assess the efficacy and safety of using montelukast sodium in combination with budesonide to treat kids with CVA. We hope that these findings can provide clinicians with more safe and effective treatment options.

## Author contributions

**Conceptualization:** Qiongyao Tang, Huizhen Lei, Jun-Yi Cao.

**Data curation:** Qiongyao Tang, Jinbing You.

**Formal analysis:** Qiongyao Tang, Jiangjiang Wang.

**Funding acquisition:** Qiongyao Tang, Jinbing You.

**Investigation:** Huizhen Lei, Jinbing You.

**Methodology:** Huizhen Lei, Jiangjiang Wang, Jun-Yi Cao.

**Resources:** Qiongyao Tang, Jinbing You.

**Software:** Huizhen Lei, Jiangjiang Wang.

**Supervision:** Huizhen Lei, Jinbing You.

**Validation:** Huizhen Lei, Jiangjiang Wang.

**Visualization:** Jinbing You, Jun-Yi Cao.

**Writing – original draft:** Qiongyao Tang, Huizhen Lei.

**Writing – review & editing:** Qiongyao Tang, Jiangjiang Wang, Jun-Yi Cao.
